# Changes in insight and outcome over the early course of first-episode psychosis. The OPTiMiSE trial

**DOI:** 10.1016/j.scog.2026.100441

**Published:** 2026-05-09

**Authors:** Javier-David Lopez-Morinigo, Covadonga M. Diaz-Caneja, Joaquin Galvañ, David Fraguas, Carmen Moreno, Gregor Berger, Stefan Leucht, Inge Winter-van Rossum, Anthony S. David, Celso Arango

**Affiliations:** a Department of Child and Adolescent Psychiatry, Institute of Psychiatry and Mental Health, Hospital General Universitario Gregorio Marañón, Instituto de Investigación Sanitaria Gregorio Marañón (IiSGM), Centro de Investigación Biomédica en Red de Salud Mental (CIBERSAM), ISCIII, School of Medicine, Universidad Complutense, Madrid, Spain; b Department of Psychiatry, Hospital Universitario del Sureste, 28500, Madrid, Spain; c Universidad Internacional de la Rioja (UNIR), Logroño, Spain; d Institute of Psychiatry and Mental Health, Hospital Clínico San Carlos, IdISSC, CIBERSAM, UCM, Madrid, Spain; e University Hospital of Psychiatry Zurich, Department of Child and Adolescent Psychiatry and Psychotherapy, Zurich, Switzerland; f Department of Psychiatry and Psychotherapy, Technical University Munich, Munich, Germany; g Department of Psychiatry, University Medical Center Utrecht, Utrecht, the Netherlands; h Division of Psychiatry, University College London, London, UK; i Hospital Universitario La Paz, IdiPAZ, School of Medicine, Universidad Autónoma de Madrid, CIBERSAM, Madrid, Spain

**Keywords:** First-episode psychosis, Insight, Outcomes, Suicide, Wellbeing, Depression, Functioning

## Abstract

**Introduction:**

Insight in psychosis has been associated with psychosis remission, treatment compliance and better functioning, but also with depression, increased suicide risk and worse subjective wellbeing. This insight paradox has not been sufficiently studied.

**Methods:**

Participants (*N* = 443) came from the first-episode psychosis OPTiMiSE trial. Insight was assessed with the clinician-rated Positive and Negative Syndrome Scale for Schizophrenia (PANSS)-G12 item. We investigated the cross-sectional associations of insight with clinical measures at baseline and at 4 weeks and the effect of insight change over the 4-week follow-up on outcomes at that point, whilst adjusting for baseline insight and other potential confounders.

**Results:**

At baseline and at week 4 better insight was cross-sectionally associated with higher rates of psychosis remission, lower illness severity and better psychosocial functioning (*p* < .001). At baseline, but not at week 4, better insight was cross-sectionally linked with more severe depressive symptomatology (*p* = .001), higher suicidality levels (*p* = .033) and worse subjective wellbeing (*p* = .022). Insight improvement was associated with higher rates of psychosis remission (*p* < .001), lower illness severity (*p* < .001) and better functioning (*p* = .014) at 4 weeks. Better baseline insight, but not insight change, was related to more severe depression (*p* < .001) and worse subjective wellbeing (*p* = .001) at 4 weeks.

**Conclusions:**

Insight was cross-sectionally associated with positive and negative clinical measures at baseline, but the latter was not replicated at 4 weeks. Improvement in insight over 4 weeks was not linked with any adverse outcome, offering reassurance to those making efforts to enhance insight early in treatment.

## Highlights

• Insight in FEP was linked with positive clinical measures at baseline and at week 4.
• At baseline, but not at 4 weeks, insight was associated with some negative outcomes.
• Change in insight over 4 weeks was not linked to any adverse outcome.
• Early interventions on poor insight may improve outcomes.

## 1 Introduction

Since the 1973 World Health Organization multicenter study, which revealed almost 9 in 10 schizophrenia patients to *lack insight* (Carpenter et al., 1973), poor insight has been assumed to be a cardinal feature of schizophrenia and related psychoses from their first presentation (Arango and Amador, 2011; Ayesa-Arriola et al., 2014). Despite this, scant attention had been paid to the study of insight until the early 1990s, when the multidimensional models of insight - illness awareness, symptom relabelling and treatment compliance - were put forward (Amador et al., 1991; David, 1990) and revived interest in the concept. Research since then has supported the multidimensionality of insight (David, 2020), its trait- and state-like properties (Parellada et al., 2011; Wiffen et al., 2010) and more recently there have been attempts to map changes in patient insight over time, especially in early psychosis (Ayesa-Arriola et al., 2014). Thus, insight has been shown to improve in response to standard treatment of psychosis over and above other symptoms. While specific evidence-based treatments for insight are lacking (Phelan and Sigala, 2022; Pijnenborg et al., 2015), metacognitive interventions have yielded promising results (Lopez-Morinigo et al., 2020).

Importantly, insight in psychosis has been linked with positive outcomes – higher insight levels, better outcomes - namely reduced psychotic symptom severity (Parellada et al., 2011; Subotnik et al., 2020), less use of coercive treatments (David, 2020) and better objectively-measured psychosocial functioning (Canal-Rivero et al., 2022; Lincoln et al., 2007; Lysaker et al., 2018). More controversially, insight gain is suggested to lead to some negative outcomes, such as more severe depressive symptoms (Belvederi Murri et al., 2015; Subotnik et al., 2020), increased risk of suicidal behaviour (López-Moríñigo et al., 2012) and poorer self-perceived quality of life or subjective wellbeing (Davis et al., 2020). Since it was proposed, this *insight paradox* (Barbalat et al., 2024; Belvederi Murri et al., 2016; Davis et al., 2020; Lysaker et al., 2007) remains subject to theoretical debate and empirical studies (Lopez-Morinigo and David, 2024). In particular, key questions remain unanswered. First, does insight really lead to some negative outcomes (e.g., lower mood, increased suicidality and/or poorer subjective wellbeing) and to what extent are these independent? That is to say, does insight predict outcomes in early psychosis when considering symptom severity, sociodemographic variables and other potential confounders? Second, does insight gain or change over the early course of psychosis have a different effect on outcomes, particularly negative ones, than when assessed at the peak of the condition? If so, does this help us better understand the insight paradox?

In order to address these issues, we analysed data from the first-episode psychosis (FEP) Optimization of Treatment and Management of Schizophrenia in Europe (OPTiMiSE) dataset (Kahn et al., 2018; Leucht et al., 2015) with a twofold aim: i) to examine the cross-sectional associations of insight with other clinical measures at baseline and at week 4; ii) to investigate the potential associations between change in insight and outcomes at week 4. In order to shed light on the insight paradox two hypotheses were tested. First, we speculated that it is insight as assessed during the height of a psychotic illness (i.e., at baseline) that shows the strongest associations with negative outcomes (depression, suicidality and worse subjective wellbeing). Second, contrary to the insight paradox assertion, we postulated that insight gain after a period of treatment and stabilisation (i.e., over the 4-week OPTiMiSE trial Phase 1), while remaining a good predictor of general clinical improvement, will not be linked with the aforementioned negative mood-related outcomes. This second hypothesis was based on our previous speculation that improving mastery over awareness-related psychological distress over the treatment course could mitigate the initial negative impact of insight on mood (Lopez-Morinigo and David, 2024).

## 2 Methods

### 2.1 Sample, study design and procedure

Data for this study came from the 27-center OPTiMiSE study, which is registered at ClinicalTrials.Gov (NCT01248195). The full methodology of the OPTiMiSE trial was detailed elsewhere (Kahn et al., 2018; Leucht et al., 2015). Briefly, inclusion criteria were: i) age: 18–40 years; ii) DSM-IV diagnosis - schizophrenia, schizophreniform disorder and schizoaffective disorder – confirmed with the Mini-International Neuropsychiatric Interview plus (Sheehan et al., 1998); iii) time period from the psychosis onset to the study inception shorter than 2 years; and iv) previous use of antipsychotics for no more than 2 weeks in the preceding year or for less than 6 weeks lifetime. Non-antipsychotic drugs (e.g. mood stabilisers and/or antidepressants) were permitted as prescribed by the treating team. Participants were treated with amisulpride (200–800 mg/day) over 4 weeks in an open-label design (OPTiMiSE Phase 1). All participants in the OPTiMiSE project provided written informed consent as approved by the local research ethics committees.

### 2.2 Insight assessment

Insight was assessed with the Positive and Negative Syndrome Scale for Schizophrenia (Kay et al., 1987) (PANSS) item G12, which measures lack of judgement and insight. For the sake of interpretation PANSS-G12 scores were reversed (1➔7, 2➔6, 3➔5, 4➔4, 5➔3, 6➔2, 7➔1) (e.g., Barrett et al., 2015) so higher (reversed) scores indicate better insight.

### 2.3 Outcome measures

We examined the effects of insight on psychosis remission, illness severity, objectively-measured psychosocial functioning, depressive symptom severity, suicidality levels and subjective wellbeing.

*Psychosis remission,* which was the primary outcome of the OPTiMiSE study (Kahn et al., 2018; Leucht et al., 2015)*,* was defined according to the criteria set out by Andreasen (Andreasen et al., 2005), that is, a maximum rating of 3 on the PANSS items P1, P2, P3, N1, N4, N6, G5, and G9. The originally required 6-month period of sustained symptom improvement (Andreasen et al., 2005) was not applied to the OPTiMiSE trial. Based on a previous review of PANSS factorial analysis studies (Wallwork et al., 2012) severity of five symptomatic dimensions was measured: positive (range: 4–23), negative (range: 6–27), disorganisation (range: 3–15), mania (range: 4–16), and depression (range: 3–16).

*Illness severity* was evaluated by means of the Clinical Global Impression (CGI) scale (Guy, 1976), i.e., higher scores, greater illness severity.

*Psychosocial functioning* was objectively measured with the Personal and Social Performance Scale (PSP) total scores, which is a clinician-based assessment of personal and social functioning (Morosini et al., 2000). Higher PSP total scores indicate better functioning.

*Depressive symptom severity* was measured by means of the Calgary Depression Scale for Schizophrenia (CDSS) (Addington et al., 1990), which is comprised of 9 items enquiring about symptoms of depression, such as hopelessness, depressed mood or guilty ideas. Each item is rated on a Likert scale from 0 to 3, yielding a total score between 0 and 27, with higher scores indicating more severe depression. Suicidality was measured using CDSS item 8, which provides a continuous score ranging from 0 (no suicidality) to 3 (previous suicide attempts).

The Subjective Wellbeing under Neuroleptics Scale (SWN) (Naber, 1995) provided a self-reported measure of *subjective wellbeing*. The SWN is 20-item scale, including 10 positive (e.g., “I find it easy to think”) and 10 negative (e.g., “I have no hope for the future”) statements, each of which is self-rated based on the degree of agreement with the statement within a 1–6 Likert scale. Individual items scores can be summed up, with higher total scores reflecting better subjective wellbeing. The SWN has shown good validity and reliability (de Haan et al., 2002).

### 2.4 Additional variables

Baseline sociodemographic and clinical data included age, sex, years of education, diagnosis (schizophrenia vs. other psychoses) and duration of untreated psychosis (DUP).

### 2.5 Statistical analysis

First, we inspected the distribution of insight levels (PANSS G12 item reversed scores) and the variables of interest at baseline and at week 4, which were normal so the mean and standard deviation (S.D.) were reported. Second, we ran a set of bivariate correlations between insight levels and other clinical measures at baseline and at week 4 (Aim 1). Third, in order to test whether ‘baseline insight’ or ‘change in insight’ from baseline to week 4 predicted outcomes at that point (Aim 2), we conducted five multivariate regression models on the aforementioned outcomes of interest at week 4: psychosis remission (Andreasen et al., 2005), illness severity (i.e., CGI total score), depressive symptom severity (i.e., CDSS total score), suicidality levels (i.e., CDSS item 8 score) and subjective wellbeing (i.e., SWN total score). Specifically, both baseline insight and insight change (among other potential confounders from bivariate analyses) were added to the models as putative predictors of the outcome variable. All analyses were performed using the Statistical Package for Social Science version 25.0 (SPSS, Inc., Chicago, IL, USA). The significance level (two-tailed) was set at *p* < .05.

## 3 Results

At baseline, *N* = 443 FES patients met the OPTiMiSE study inclusion criteria (Intention-to-Treat sample) and had depression and insight data available. Mean age was 24.7 years (S.D. 5.7), *n* = 132 (29.8%) were females and mean education years was 12.3 (S.D. 2.9). Out of N = 443 participants, *n* = 225 (50.8%) were diagnosed with schizophrenia, with a median DUP of 4 months. The 4-week Phase 1 was completed by *n* = 371 participants (83.7%).

**Fig. 1:**
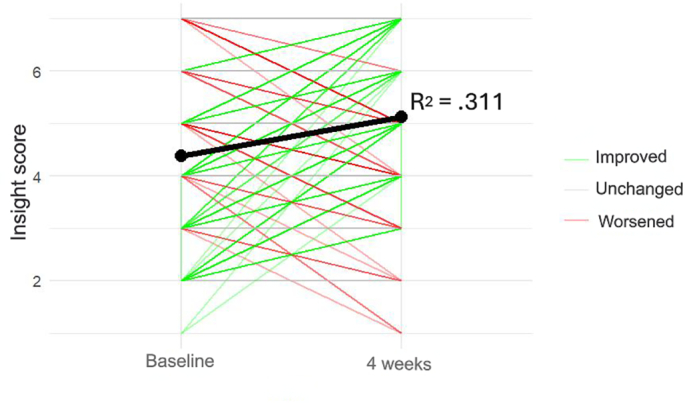
Insight changes over the 4-week follow-up.

At baseline the mean reversed PANSS G12 score was 4.38 ± 1.40, while at week 4 reversed PANSS G12 score mean was 5.12 ± 1.35 (*p* < .001). More specifically, from baseline to week 4, most patients (*n* = 199, 53.8%) showed improved insight (i.e., reversed PANSS G12 score at week 4 was higher than at baseline). In contrast, insight did not change in *n* = 127 subjects (34.3%), while *n* = 44 participants (11.9%) exhibited a decrease in insight (i.e., reversed PANSS G12 score at week 4 was lower than at baseline) over this 4-week period. These results are illustrated in the scatter plot shown in Fig. 1.

Baseline insight values significantly influenced (*p* < .001) the proportion of patients who showed improvement in insight, with lower baseline insight associated with a greater proportion of improvers. For each (reversed) baseline PANSS G12 item score, the proportion of insight improvers was as follows: 1 (100%), 2 (88.9%), 3 (79.6%), 4 (61.0%), 5 (44.9%), 6 (33.3%), and 7 (0%).

See Table 1 for details.

**Table 1:** Insight and outcomes values at baseline and at week 4.

	Baseline	Week 4	Statistic	*p*
PANSS G12 reverse	4.38 ± 1.40	5.12 ± 1.35	*t* = 10.73	< 0.001

*Outcomes*				
PANSS Total score	77.7 ± 18.8	58.2 ± 18.5	*t* = −20.38	< 0.001
PANSS Positive	12.4 ± 13.0	10.1 ± 3.8	*t* = −15.51	< 0.001
PANSS Negative	15.7 ± 6.6	13.4 ± 5.9	*t* = −7.97	< 0.001
PANSS Disorganisation	8.6 ± 3.1	6.3 ± 2.7	*t* = −15.49	< 0.001
PANSS Mania	7.6 ± 3.3	5.7 ± 2.6	*t* = −11.44	< 0.001
PANSS Depression	7.9 ± 3.3	5.8 ± 2.8	*t* = −12.50	< 0.001
Remission	n = 46 (10.4%)	*n* = 249 (67.3%)		< 0.001
CDSS Total score	4.5 ± 4.6	2.8 ± 3.9	*t* = −7.73	< 0.001
CDSS 8 (suicidality)	0.3 ± 0.6	0.1 ± 0.4	*t* = −5.89	< 0.001
CGI total score	4.5 ± 0.9	3.4 ± 1.1	*t* = −17.89	< 0.001
PSP total score	48.7 ± 15.5	61.8 ± 16.1	*t* = 13.67	< 0.001
SWN total score	81.5 ± 16.0	89.5 ± 15.6	*t* = 10.27	< 0.001

PANSS: Positive and Negative Syndrome Scale for Schizophrenia (Kay et al., 1987). CDSS: Calgary Depression Scale for Schizophrenia (Addington et al., 1990). CGI: Clinical Global Impression (Guy, 1976). PSP: Personal and Social Performance Scale (Morosini et al., 2000). SWN: Subjective Wellbeing under Neuroleptics (Naber, 1995).

### 3.1 Cross-sectional associations of insight with outcomes at baseline and at week 4

At baseline, those in remission (Andreasen et al., 2005) had better insight than non-remitters (5.1 ± 1.2 vs. 4.3 ± 1.4, *p* < .001). Also, better insight was associated with lower illness severity (*r* = −0.34, *p* < .001), better psychosocial functioning (*r* = 0.21, *p* < .001), more severe depressive symptomatology (*r* = 0.16, *p* < .001), higher suicidality levels (*r* = 0.10, *p* = .033) and worse subjective wellbeing (*r* = −0.11, *p* = .022).

At week 4, those in remission had higher insight levels than non-remission participants (5.5 ± 1.2 vs. 4.4 ± 1.3, *p* < .001). Also, better insight was correlated with lower illness severity (*r* = −0.44, *p* < .001) and better psychosocial functioning (*r* = −0.37, *p* < .001). See Table 2 for further details.

**Table 2:** Cross-sectional associations of insight with outcome measures.

	Cross-sectional associations of insight levels with outcome values					
	At Baseline (n = 443)			At Week 4 (n = 371)		
*Binary outcomes*	*Mean ± SD*		*p*	*Mean ± SD*		*p*
Remission	R	NR		R	NR	
	n = 46	*n* = 397		n = 249	*n* = 122	
	5.1 ± 1.2	4.3 ± 1.4	< 0.001	5.5 ± 1.2	4.4 ± 1.3	< 0.001
*Continuous outcomes*	*r*		*p*	*r*		*p*
CGI total score	−0.34		<0.001	−0.44		< 0.001
PSP total score	0.21		<0.001	0.37		< 0.001
CDSS total score	0.16		0.001	−0.0.06		0.23
CDSS 8 score	0.10		0.033	−0.06		0.27
SWN total score	−0.11		0.030	0.08		0.14

R: In remission (Andreasen et al., 2005). NR: In non-remission. D: Discontinued treatment. ND: Did not discontinue treatment.

CDSS: Calgary Depression Scale for Schizophrenia (Addington et al., 1990). CGI: Clinical Global Impression (Guy, 1976).

PSP: Personal and Social Performance Scale (Morosini et al., 2000). SWN: Subjective Wellbeing under Neuroleptics (Naber, 1995).

### 3.2 Change in insight and outcomes at week 4

#### 3.2.1 Positive outcomes

##### 3.2.1.1 Regression on psychosis remission

Both baseline insight (OR = 2.04, 95% CI 1.60–2.59, *p* < .001) and change in insight (OR = 2.05, 95% CI 1.58–2.66, *p* < .001) were linked with psychosis remission – better insight, higher rates of remission -. Additionally, (older) age, diagnosis of schizophrenia (vs. other psychoses) – schizophrenia, lower rates of baseline remission -, and mood – more severe depression, lower rates of remission- were associated with higher rates of psychosis remission at week 4 (see Table 3).

**Table 3:** Multivariable logistic regression model on psychosis remission at week 4.

	B	SE	Wald	p	Exp (B)	95% CI
Age	0.051	0.025	4.195	0.041	1.053	1.002–1.105
Sex	0.443	0.335	1.743	0.187	1.557	0.807–3.005
Education level	0.003	0.049	0.003	0.957	1.1003	0.911–1.104
DUP	−0.007	0.024	0.082	0.775	0.993	0.948–1.041
Diagnosis (SZ)	−0.922	0.327	7.969	0.005	0.398	0.210–0.754
Baseline Insight	0.712	0.122	34.008	<0.001	2.038	1.604–2.588
Insight change	0.720	0.133	29.376	<0.001	2.054	1.583–2.665
Depression	−0.168	0.037	21.036	<0.001	0.846	0.787–0.908

DUP: duration of untreated psychosis. SZ: schizophrenia.

##### 3.2.1.2 Regression on illness severity

Change in insight (*p* < .001), but not baseline insight, was linked with illness severity at week 4 (i.e., CGI score) -better insight, lower illness severity-, accounting for 3% of the variance in illness severity at that point, which was also associated with DUP, psychotic and depressive symptom severity (Table S1, online supplementary material).

##### 3.2.1.3 Regression on psychosocial functioning

Change in insight (*p* = .014), but not baseline insight, emerged as predictor of psychosocial functioning (i.e., PSP total score), explaining 1% of the variance. Also, DUP, illness severity, psychotic and depressive symptom severity were linked to functioning at week 4 (Table S2, online supplementary material).

#### 3.2.2 Negative outcomes

##### 3.2.2.1 Regression on depressive symptom severity

Baseline insight, but not change in insight, explained 4.9% (*p* < .001) of the variance in depressive symptom severity, as measured with the CDSS total score – better insight, more severe depression – at week 4, (Table 4).

**Table 4:** Predictors of depressive symptomatology (CDSS) at week 4.

Blocks	R^2^ ch.	F ch.	p
1) Sociodemographics	0.010	1.043	0.374
2) DUP	< 0.001	0.080	0.778
3) SZ (vs. others)	< 0.001	0.011	0.918
4) CGI total score	0.127	46.572	< 0.001
5) PANSS Total score	0.039	4.998	0.002
6) Baseline Insight	0.049	19.959	<0.001
7) Insight Change	0.001	0.421	0.517
MODEL	21.5%		

CDSS: Calgary Depression Scale for Schizophrenia (Addington et al., 1990). DUP: duration of untreated psychosis. SZ: schizophrenia. CGI: Clinical Global Impression (Guy, 1976). PANSS: Positive and Negative Syndrome Scale for Schizophrenia (Kay et al., 1987).

##### 3.2.2.2 Regression on suicidality

Neither baseline insight nor insight change were related to suicidality levels at week 4 (Table S3, online supplementary material).

##### 3.2.2.3 Regression on subjective wellbeing

Baseline insight explained 2.2% (*p* < .001) of the variance in subjective wellbeing at week 4, whereas change in insight over this 4-week period was not linked with subjective wellbeing at that point (Table 5).

**Table 5:** Predictors of subjective wellbeing (SWN) at week 4.

Blocks	R^2^ ch.	F ch.	p
1) Sociodemographics	0.018	1.859	0.137
2) DUP	0.035	11.174	0.001
3) SZ (vs. others)	0.002	0.766	0.382
4) CGI total score	0.170	65.891	< 0.001
5) PANSS total score	0.011	1.495	0.216
6) CDSS total score	0.184	94.071	< 0.001
7) Baseline Insight	0.022	11.799	0.001
8) Insight Change	< 0.001	0.077	0.781
MODEL	41.1%		

SWN: Subjective Wellbeing under Neuroleptics (Naber, 1995). DUP: Duration of untreated psychosis. SZ: schizophrenia. PANSS: Positive and Negative Syndrome Scale for Schizophrenia (Kay et al., 1987). CDSS: Calgary Depression Scale for Schizophrenia (Addington et al., 1990). CGI: Clinical Global Impression (Guy, 1976).

## 4 Discussion

### 4.1 Main findings

**Fig. 2:**
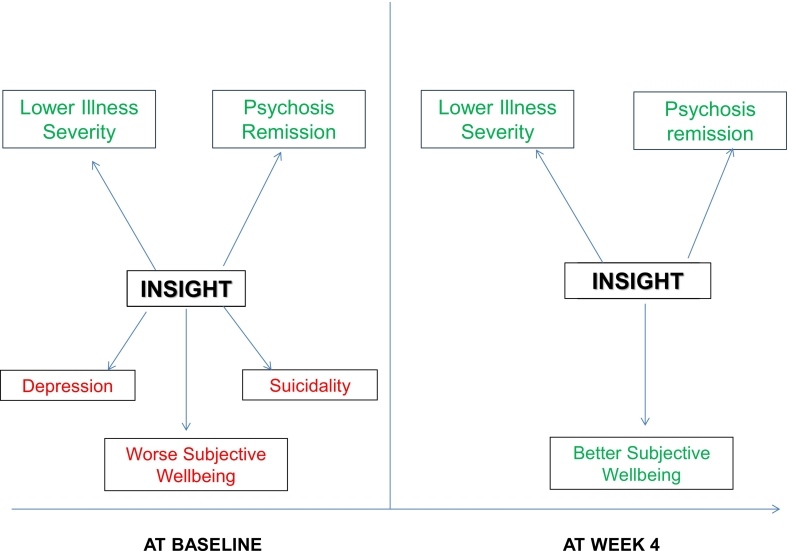
Directed Acyclic Graph showing the associations of insight with positive (in green) and negative (in red) outcomes at baseline and at week 4.

We retrieved data from the FEP OPTiMiSE study in order to better understand the so-called *insight paradox* in the early stages of psychosis. Three main findings emerged from the analyses. First, we replicated the cross-sectional associations of better insight with positive outcomes (lower illness severity, higher rates of psychosis remission and better functioning) both at baseline and at 4 weeks. Moreover, improvement in insight over the 4-week follow-up period was linked with these positive outcomes at that point. Second, baseline insight was cross-sectionally associated with more severe depression, higher suicidality levels and worse subjective wellbeing, thus providing partial support for the insight paradox. However, these associations were not observed at 4 weeks. Third, this insight change over the follow-up was not associated with any negative outcome at 4 weeks. Taken together, these findings seem to challenge the simple version of the insight paradox. Alternatively, it could be argued that, at least in some individuals, insight improves rapidly in parallel with treatment-related remission of psychosis, as discussed further below. For an overview of results and the inter-relationships between variables, we have constructed a Directed Acyclic Graph (DAG) (see Fig. 2).

### 4.2 Cross-sectional associations of insight at baseline and at week 4

The so-called insight paradox (Lysaker et al., 2007) posits that insight in psychosis is associated with positive (higher rates of psychosis remission, lower illness severity and better functioning) and negative (depression, suicidality and poorer self-perceived quality of life) outcomes, although the latter requires further theoretical debate and more empirical research (Lopez-Morinigo and David, 2024). We speculated that this is only the case for cross-sectional associations, on which we based our first hypothesis, as supported by results. In particular, at baseline better insight was linked with higher rates of remission, lower illness severity and better functioning, but also with more severe depressive symptoms, higher suicidality levels and poorer subjective wellbeing both at baseline and at 4 weeks.

More controversially, the insight paradox predicted that insight may lead to some degree of demoralisation, including lower mood (Amore et al., 2020; Belvederi Murri et al., 2015; Subotnik et al., 2020), higher suicide risk (López-Moríñigo et al., 2012) and worse self-appraised quality of life (Davis et al., 2020). Consistent with this, we expected to find better insight to be cross-sectionally associated with greater depressive symptom severity, higher suicidality levels and poorer subjective wellbeing in the OPTiMiSE study. While baseline data supported this, no such associations were replicated at 4 weeks. In this respect, it is worth noting that a previous systematic review (López-Moríñigo et al., 2012) and a meta-analysis (Belvederi Murri et al., 2015) suggested that previous cross-sectional studies showing an association of insight with depression (Amore et al., 2020) and suicidality (López-Moríñigo et al., 2012) may be subject to selection bias and recall bias. Thus, according to the Bradford Hill's causality criteria (Hill, 1965) no outcome variable (i.e., depression and/or suicidal behaviour) can precede a putative predictor (i.e., insight). Moreover, based on the depressive realism model more depressed subjects and/or suicide attempters may more easily recall previous adverse events such as having a mental illness, hence giving rise to higher insight scores at the assessment (Ghaemi and Rosenquist, 2004), thus introducing a recall bias. These findings suggest a bidirectional relationship between insight and depression, which may be considered as “two sides of the same coin”. As a result, an overfocus on cross-sectional studies may have contributed to reaching the (wrong) conclusion that insight (development) leads to depression/suicidality (López-Moríñigo et al., 2012). Of concern, we speculated that this notion may explain why some clinicians appear to be tempted to counsel against enhancing insight (Lopez-Morinigo and David, 2024).

From a clinical perspective, it should be noted that third party unmeasured variables, including patient context and background, socio-cultural issues or the patient-doctor relationship (Ariyo et al., 2022; Belvederi Murri and Amore, 2019) are likely to influence the association between insight and depression/suicidality. To address this issue, future studies adopting both quantitative and qualitative approaches are warranted.

### 4.3 Change in insight and outcome

First of all, we replicated the positive impact of insight gain on general clinical improvement, hence in full agreement with previous longitudinal studies in first-episode psychosis (FEP) (Ayesa-Arriola et al., 2018; Canal-Rivero et al., 2022; Cuesta et al., 2011) and in schizophrenia (Capdevielle et al., 2021).

With a focus on the potential negative effects of insight, we tested the hypothesis that insight gain over the early course of psychosis is not necessarily linked to lower mood, higher risk of suicidal behaviour or worse self-perceived quality of life and subjective wellbeing. In particular, this hypothesis was based on our speculation that improving mastery over awareness-related psychological distress may relieve low mood and decrease internalised stigma (Lopez-Morinigo and David, 2024), thus mitigating the initially demoralising aspects of insight development. Supporting this, we found no association between insight gain over the 4-week follow-up period and depression, suicidality or (worse) subjective wellbeing, whereas baseline insight itself predicted depression and worse subjective wellbeing. These findings were consistent with earlier FEP (Barrett et al., 2015) and schizophrenia (Bourgeois et al., 2004) longitudinal intervention studies and more recent cross-sectional schizophrenia (Massons et al., 2017; Roux et al., 2018) and FEP cohort studies (Ayesa-Arriola et al., 2018; Lopez-Morinigo et al., 2019), all of which failed to establish a direct link between insight development and higher risk of depression/suicidality. Certainly, there seems to be a complex dynamic interplay between insight, depression/suicidality and subjective wellbeing, which might be mediated by self-esteem (Yanos et al., 2021) and internalised stigma (Belvederi Murri et al., 2016; Chio et al., 2018; Eliasson et al., 2021; Lysaker et al., 2007; Yang et al., 2020). Regrettably, neither self-esteem nor internalised stigma was assessed in the OPTiMiSE project. On the other hand, a 12-month follow-up schizophrenia cohort study from France revealed a direct detrimental effect of insight on depression, quality of life and suicidality (Ehrminger et al., 2019).

From the subjective patient's perspective, in addition to the concept of quality of life (QoL), in our study we evaluated so-called subjective wellbeing (de Haan et al., 2002). Of relevance, better insight predicted worse subjective wellbeing at baseline, but not at 4 weeks. Although insight appears to affect QoL self-appraisals (Kim et al., 2015), depressive symptoms may play a mediating role in the effect of illness awareness on QoL (Margariti et al., 2015), which requires future investigation. Most importantly, no psychosocial intervention has been demonstrated to improve QoL in schizophrenia spectrum disorders to date (Puolakka and Pitkänen, 2019).

Furthermore, insight change over the 4-week follow-up did not result in worse (or better) subjective wellbeing, which conflicted with a 2020 meta-analysis showing global insight to have a small negative effect on QoL – better insight, worse QoL – (Davis et al., 2020). Of note, poor insight patients tend to more positively self-perceive their QoL compared with their treating physician, hence the type of assessment of QoL (assessor-rated versus self-report) may affect the relationship between insight and QoL (Lysaker et al., 2022). While this study partially supports the insight paradox notion (Lysaker et al., 2022), this is incompatible with the well-established positive short- (Lee et al., 2019; van Baars et al., 2013) and long-term effect of insight on functioning (Canal-Rivero et al., 2022), thus highlighting the fundamental difference between objective (e.g., PSP) and subjective (e.g., SWN) measures of functional outcome. Moreover, insight improvement was linked to higher scores on a subjective measurement of one's wellbeing, such as the SWN. Contrary to the commonly held view among clinicians, insight development may therefore improve subjective wellbeing and QoL. More specifically, early intervention on poor insight may contribute to better short-term patient outcome. This may be particularly relevant for individuals with poorer baseline insight, who have greater potential for improvement, as supported by our results. Hence, the key question is not whether improving insight is inherently “good” or “bad” (Lopez-Morinigo and David, 2024), but rather how such changes should be understood in context. A more patient-centred approach should therefore be adopted, considering the individual as a whole, including their psychosocial circumstances (Lysaker et al., 2018).

### 4.4 Strengths and limitations

To the best of our knowledge, this is the first study in examining change in insight and outcome over the early course of psychosis in an antipsychotics clinical trial. Variables and outcomes were adequately measured with validated instruments. Of relevance, the longitudinal design of the OPTiMiSE allowed us to examine the relationship between insight changes and outcome, although future long-term large-scale clinical trials are warranted, as discussed further below.

However, several limitations of this study should be acknowledged. First, owing to the methodology of the OPTiMiSE project, which was not originally designed to test this study hypotheses, subsamples over the trial phases may have lacked sufficient to detect smaller effects. In particular, the inclusion of the OPTiMiSE Phase 2 and Phase 3 subsamples may have incorporated a selection bias since only non-remission patients at the end of Phase 1 and Phase 2 were invited to take part in Phase 2 and Phase 3, respectively, hence, those with more severe symptomatology and poorer insight. In this respect, it should be noted that being in remission at baseline according to the criteria set out by Andreasen and colleagues (Andreasen et al., 2005) was not an OPTiMiSE exclusion criterion. Second, all participants fulfilled the inclusion/exclusion criteria for the OPTiMiSE trial to which they consented, which may have excluded those with poorer insight, thus limiting the generalisablity of results. Third, the proportion of the variance in outcomes explained by the models were relatively small. Other non-tested variables, such as premorbid personality and adjustment, should therefore be included in future studies. More specifically, self-esteem (Yanos et al., 2021) and internalised stigma (Belvederi Murri et al., 2016; Chio et al., 2018; Eliasson et al., 2021; Lysaker et al., 2007; Yang et al., 2020), none of which was assessed in this study, were reported to mediate the relationship between insight and depression/suicidality. Fourth, insight was assessed unidimensionally with the PANSS item which cannot capture its multidimensionality, i.e., individual insight dimensions may have different effects on outcomes. Moreover, PANSS G12 is an ordinal single item that, for the purposes of the analyses, was treated as a continuous variable. Caution is therefore needed when interpreting the study results, although these were supported by preliminary sensitivity analyses with the OPTiMiSE Phase 2 subsample (available upon request). This noted, both the PANSS insight item (Sanz et al., 1998) and a recently validated unidimensional proxy measure (López-Díaz et al., 2022) were showed to strongly correlate with multidimensional insight scales such as the Scale to Assess Unawareness of Mental Disorder (SUMD) (Amador et al., 1993) and the Schedule for Assessment of Insight (SAI) (David, 1990) and its expanded version (SAI-E) (David and Kemp, 1997). Also, psychotic symptom severity and insight were both unblinded assessed with the same instrument (PANSS) which may have influenced each other. Further to this, both subjective (e.g., SWN) and objective (e.g., PSP) measures were included for the reasons outlined above, encompassing both self-reported and rater-rated scales, which should be considered compositional data rather than causal endpoints (Tomova et al., 2025). Fifth, although the relatively short follow-up period of the OPTiMiSE trial Phase 1 did not permit us to study longer-term insight changes and outcomes, this 4-week period was in line with the length of stay for adult acute inpatient psychiatric wards in most Western countries (OECD, 2023). Furthermore, the study design (i.e., two assessments over a 4-week period) did not allows us to examine non-linear changes, trajectories, lagged dynamics, random slopes or time-varying confounding*.* Finally, concomitant treatments were permitted, although only a small proportion of participants in the OPTiMiSE project were receiving other medications; for instance, *n* = 46 participants (less than 10%) were on antidepressants prior to the study inception (Lopez-Morinigo et al., 2025). Since previous studies failed to find between-treatment differences in insight (Phelan and Sigala, 2022) and all Phase 1 participants were treated with amisulpride, such a minimal exposure to concomitant treatments was unlikely to have affected our results. This said, future clinical trials of antipsychotics (and other psychiatric medications) should include insight measures as outcome.

### 4.5 Implications on clinical practice and future research

Although the cross-sectional associations of baseline insight with positive and negative outcomes provided some support for the insight paradox, insight improvement over the early course of psychosis was not linked with any detrimental effect. From a patient-centred approach, however, any treatment for enhancing insight should be delivered with strategies to improve mastery over awareness-related psychological distress, thus relieving low mood and decreasing internalised stigma (Yang et al., 2020). To this end, interventions targeting metacognitive skills by enhancing self-reflectiveness can now be evidence-based recommended (García-Mieres et al., 2020; Lopez-Morinigo et al., 2020). However, future long-term large-scale clinical trials should demonstrate whether an insight-improving intervention may reduce risk of depression/suicidality and improve QoL or subjective wellbeing. From the other way round, randomised controlled trials of treatments for depression in psychosis may test whether improved mood affects insight. Alternatively, insight may improve in parallel with remission of psychosis, which may reduce the need for early efforts at addressing impaired insight.

## CRediT authorship contribution statement

**Javier-David Lopez-Morinigo:** Writing – review & editing, Writing – original draft, Software, Investigation, Formal analysis. **Covadonga M. Diaz-Caneja:** Writing – review & editing, Supervision, Conceptualization. **Joaquin Galvañ:** Writing – review & editing, Software. **David Fraguas:** Writing – review & editing, Supervision, Methodology, Conceptualization. **Carmen Moreno:** Writing – review & editing. **Gregor Berger:** Writing – review & editing. **Stefan Leucht:** Writing – review & editing. **Inge Winter-van Rossum:** Writing – review & editing. **Anthony S. David:** Writing – review & editing, Investigation, Conceptualization. **Celso Arango:** Writing – review & editing, Investigation, Conceptualization.

## Declaration of competing interest

The authors have no competing interests to disclose in relation to the study subject.

## Data Availability

Data supporting these results are available upon reasonable request to the corresponding author, provided the OPTiMiSE dataset access policy is complied with.

## Appendix A Supplementary data

Table S1 Predictors of illness severity (CGI) at week 4

Table S2 Predictors of psychosocial functioning (PSP) at week 4

Table S3 Predictors of suicidality (CDSS-8) at week 4
