# Supplementary material for: Changes in insight and outcome over the early course of first-episode psychosis. The OPTiMiSE trial
Source: Schizophr Res Cogn. 2026 May 9;45:100441. doi: 10.1016/j.scog.2026.100441 (PMC13187536; doi:10.1016/j.scog.2026.100441)
Supplement: Table S1 — Predictors of illness severity (CGI) at week 4 [file mmc1.docx]

| **Table S1. Predictors of illness severity (CGI) at week 4** | | | |
| --- | --- | --- | --- |
| Blocks | R^2^ ch. | F ch. | p |
| 1) Sociodemographics | 0.020 | 2.181 | .090 |
| 2) DUP | 0.057 | 19.518 | < .001 |
| 3) SZ (vs. others) | 0.001 | 0.330 | .566 |
| 5) PANSS total score | 0.382 | 74.235 | < .001 |
| 6) CDSS total score | 0.051 | 32.532 | < .001 |
| 7) Baseline Insight | 0.003 | 2.015 | .157 |
| 8) Change Insight | 0.030 | 20.651 | < .001 |
| MODEL | 52.0 |  |  |
| DUP: Duration of untreated psychosis. SZ: schizophrenia. PANSS: Positive and Negative Syndrome Scale for Schizophrenia (Kay et al., 1987). CDSS: Calgary Depression Scale for Schizophrenia (Addington et al., 1990). CGI: Clinical Global Impression (Guy, 1976). | | | |
