# Supplementary material for: Changes in insight and outcome over the early course of first-episode psychosis. The OPTiMiSE trial
Source: Schizophr Res Cogn. 2026 May 9;45:100441. doi: 10.1016/j.scog.2026.100441 (PMC13187536; doi:10.1016/j.scog.2026.100441)
Supplement: Table S2 — Predictors of psychosocial functioning (PSP) at week 4 [file mmc2.docx]

| **Table S2. Predictors of psychosocial functioning (PSP) at week 4** | | | |
| --- | --- | --- | --- |
| Blocks | R^2^ ch. | F ch. | *p* |
| 1) Sociodemographics | 0.020 | 2.171 | .091 |
| 2) DUP | 0.028 | 9.381 | .002 |
| 3) SZ (vs. other) | 0.001 | 0.288 | .592 |
| 4) CGI | 0.421 | 247.839 | < .001 |
| 5) PANSS total score | 0.022 | 4.365 | .005 |
| 6) CDSS | 0.011 | 6.943 | .009 |
| 7) Baseline Insight | 0.001 | 0.389 | .533 |
| 8) Insight Change | 0.010 | 6.141 | .014 |
| MODEL | 49.2% |  |  |
| PSP: Personal and Social Performance Scale (Morosini et al., 2000). DUP: Duration of untreated psychosis. SZ: schizophrenia. CGI: Clinical Global Impression (Guy, 1976). PANSS: Positive and Negative Syndrome Scale for Schizophrenia (Kay et al., 1987). CDSS: Calgary Depression Scale for Schizophrenia (Addington et al., 1990). | | | |
