# Supplementary material for: Changes in insight and outcome over the early course of first-episode psychosis. The OPTiMiSE trial
Source: Schizophr Res Cogn. 2026 May 9;45:100441. doi: 10.1016/j.scog.2026.100441 (PMC13187536; doi:10.1016/j.scog.2026.100441)
Supplement: Table S3 — Predictors of suicidality (CDSS-8) at week 4 [file mmc3.docx]

| **Table S3. Predictors of suicidality (CDSS-8) at week 4** | | | |
| --- | --- | --- | --- |
| Blocks | R^2^ ch. | F ch. | p |
| 1) Sociodemographics | 0.017 | 1.859 | .136 |
| 2) DUP | 0.009 | 2.917 | .089 |
| 3) SZ (vs. others) | 0.001 | 0.209 | .648 |
| 4) CGI | 0.032 | 10.672 | <.001 |
| 5) PANSS total score | <0.001 | 0.004 | .951 |
| 6) CDSS total score | 0.247 | 115.410 | <.001 |
| 7) Baseline Insight | <0.001 | 0.061 | .806 |
| 8) Insight Change | <0.001 | 0.004 | .951 |
| MODEL | 27.9% |  |  |
| CDSS: Calgary Depression Scale for Schizophrenia (Addington et al., 1990). DUP: Duration of untreated psychosis. SZ: schizophrenia. CGI: Clinical Global Impression (Guy, 1976). PANSS: Positive and Negative Syndrome Scale for Schizophrenia (Kay et al., 1987). | | | |
